# Metasurface-Based Image Classification Using Diffractive Deep Neural Network

**DOI:** 10.3390/nano14221812

**Published:** 2024-11-12

**Authors:** Kaiyang Cheng, Cong Deng, Fengyu Ye, Hongqiang Li, Fei Shen, Yuancheng Fan, Yubin Gong

**Affiliations:** 1 International School of Microelectronics, Dongguan University of Technology, Dongguan 523808, China; chengky@dgut.edu.cn (K.C.); 221115193@dgut.edu.cn (C.D.); yefy@dgut.edu.cn (F.Y.); ybgong@uestc.edu.cn (Y.G.); 2 College of Electronic and Information Engineering, Tongji University, Shanghai 200092, China; hqlee@tongji.edu.cn; 3 The Institute of Dongguan Tongji University, Dongguan 523808, China; 4 Key Laboratory of Light Field Manipulation and Information Acquisition, Ministry of Industry and Information Technology and School of Physical Science and Technology, Northwestern Polytechnical University, Xi’an 710129, China; 5 National Key Laboratory on Vacuum Electronics, University of Electronic Science and Technology of China (UESTC), Chengdu 610054, China

**Keywords:** metasurfaces, diffractive deep neural network, image classification

## Abstract

The computer-assisted inverse design of photonic computing, especially by leveraging artificial intelligence algorithms, offers great convenience to accelerate the speed of development and improve calculation accuracy. However, traditional thickness-based modulation methods are hindered by large volume and difficult fabrication process, making it hard to meet the data-driven requirements of flexible light modulation. Here, we propose a diffractive deep neural network (D^2^NN) framework based on a three-layer all-dielectric phased transmitarray as hidden layers, which can perform the classification of handwritten digits. By tailoring the radius of a silicon nanodisk of a meta-atom, the metasurface can realize the phase profile calculated by D^2^NN and maintain a relative high transmittance of 0.9 at a wavelength of 600 nm. The designed image classifier consists of three layers of phase-only metasurfaces, each of which contains 1024 units, mimicking a fully connected neural network through the diffraction of light fields. The classification task of handwriting digits from the ‘0’ to ‘5’ dataset is verified, with an accuracy of over 90% on the blind test dataset, as well as demonstrated by the full-wave simulation. Furthermore, the performance of the more complex animal image classification task is also validated by increasing the number of neurons to enhance the connectivity of the neural network. This study may provide a possible solution for practical applications such as biomedical detection, image processing, and machine vision based on all-optical computing.

## 1. Introduction

Analog optical computing [1,2,3,4,5,6], as an alternative computing architecture, has shown great potential with its inherent parallelism, energy efficiency, and low latency. Compared with digital systems, the analog approaches are particularly capable of dealing with massive computational tasks, for instance, in the fields of biological/medical imaging, object classification, astronomical observation, and machine vision [1,7,8,9,10,11]. Despite its potential for various applications, designing a computational optical network is intricate and time-consuming, since the designer has to meticulously select the parameters of the whole structure and nonlinear relationship, making it less straightforward and more unpredictable to find the optimal solution. Recently, with significant advances in computational power, it has become feasible to automatically design optical computing devices based on deep neural network algorithms [12,13]. These advancements allow researchers to leverage the powerful capabilities of deep learning [14,15] to model and optimize optical systems in a more efficient way.

The computer-assisted inverse design of optical devices is an active field [16,17,18,19]; here, we focus on an all-optical diffractive deep neural network (D^2^NN) framework [20,21,22,23,24,25], where the connections of neurons are formed by the physical reality of the propagation of light through different diffractive optical elements (DOEs). By using conventional deep learning methods such as gradient descent and error back-propagation, the phase and/or amplitude of each optical element can be determined according to the Huygens–Fresnel principle. Nevertheless, in order to achieve the arbitrary and precise modulation capabilities calculated by artificial intelligence, conventional thickness-based modulators cannot meet the requirements as they suffer the drawbacks of being bulky, inefficient, and having low resolution. To overcome these challenges, a kind of two-dimensional artificial electromagnetic (EM) material called a metasurface [26,27,28,29,30,31,32] has been introduced as a powerful tool which can enable us to engineer the spatial distribution of amplitude, phase, and polarization response with subwavelength resolution. Their ability to impart abrupt phase shifts on a scattered wave allow for the creation of tailored wavefronts and the realization of various functionalities such as negative refraction, flat lenses [33,34,35], EM wave absorber, and a signal processing system.

Table 1 shows the computational performance of different D^2^NN models. As shown in Table 1, diffractive optical neural networks (DONNs [36]) and multi-wavelength diffractive deep neural networks (Multi-wavelength D^2^NNs [37]) achieve high accuracy on the MNIST dataset using the single-wavelength and multi-wavelength approaches, respectively. The single-layer diffractive neural network (SL-DNN [38]) model improved the expressive capability of single-layer structures by controlling the Fresnel number, resulting in higher accuracy. The diffractive deep neural networks at visible wavelengths (Visible Light D^2^NN [39]) model introduced a general theory that included a correction formula, quantifying the match between theoretical design and manufacturing system performance (wavelength, neuron size, and manufacturing constraints). The reconfigurable digital all-optical diffractive neural network (R-ODNN [40]) model proposed a reconfigurable structure, enabling the network to be reconfigurable, digital, and non-volatile. In this article, based on the findings of other researchers, in order to ensure an accuracy rate of over 90% while reducing the overall size of the D^2^NN structure, we propose a metasurface based image classifier using a diffractive neural network algorithm working at wavelength of 600 nm. Three all-dielectric phased transmitarrays [41,42,43,44] are cascaded as hidden layers, where the phase distributions of the metasurfaces are calculated by phase-only D^2^NN [45] through training a large number of samples in the handwritten digit database. The incident light passing through the metal mask will carry image information and eventually be focused at a specific location after being modulated by the multi-layer scatterers, simulating the fully connected neural network. By adjusting the radius of the silicon nanorods, the meta-atom can achieve a complete 2π phase shift coverage range and maintain high transmittance at a wavelength of 600 nm. Through numerical calculation and verified by the full wave simulation, it is shown that the proposed metasurface image classifier can effectively control the phase of light and achieve an overall accuracy of 96.2% for six-class digit image classification. We believe the proposed system is a potential candidate in fields such as optical device design and defect detection.

## 2. Materials and Methods

**Figure 1:**
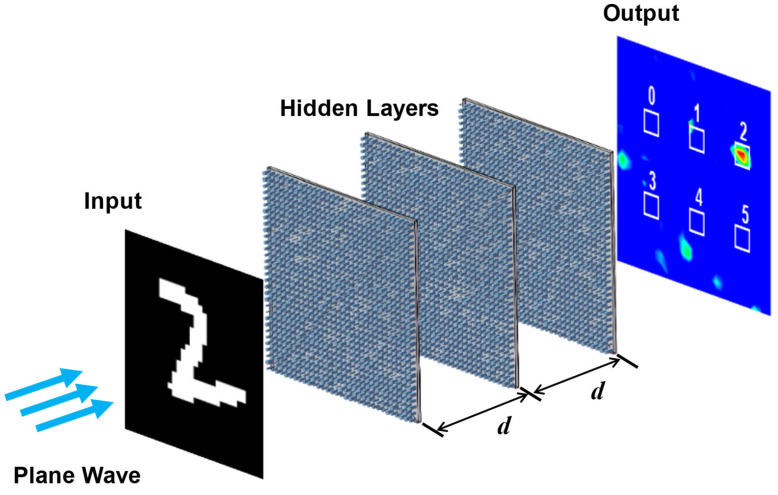
Schematic diagram of the proposed image classifier. An incident plane wave passes through the input plane carrying the information of handwriting digits and undergoes three-layer phased transmitarray which provide layer-by-layer phase modulation with spacing of *d* = 6.5 μm. After propagating the entire system, light will be focused at a specific location of the output plane corresponding to different digits. From the light intensity distribution pattern, it can be inferred that the target being measured at this moment is the handwritten digit ‘2’.

The proposed three-layer phase-only D^2^NN architecture is illustrated in Figure 1. The samples used for learning and training are derived from the MNIST dataset with handwritten digits from ‘0’ to ‘5’. Unlike traditional artificial neural networks, which can directly extract pixel information from the image samples, here, in order to facilitate the optical implementation, we make some adjustments to the original samples where the grayscale values of digit images are binarized into 0 and 1 with a threshold of 0.2. Pixel values of 0 and 1 indicate the transparent and opaque statuses, respectively, which can be realized by placing a perforated metal plate at the input plane. Then, we pad zeros to the original images (28 × 28 pixels) in order to make it consistent with the pixel size of the diffraction layers (32 × 32 pixels). Each diffraction layer consists of 32 × 32 meta-atoms (corresponding to neurons in D^2^NN) with an interlayer spacing of *d* = 6.5 μm.

### 2.1. Algorithm of D^2^NN

During the training of the diffractive neural network model, data enter the network through the input layer and propagate forward to produce an output. The weights of the neurons are optimized through an error backpropagation algorithm. According to the Rayleigh–Sommerfeld diffraction formula [46], each point on the diffraction surface can be regarded as a secondary spherical wavelet source, and the spatial distribution of a light field is the result of a coherent superposition of the wavelet sources after diffraction propagation. The diffraction propagation formula for the neurons is expressed as follows:

(1) $$\left\{\begin{array}{l} w_{i}^{l}(x,y,z)=\frac{z-z_{i}}{r^{2}}(\frac{1}{2\pi r}+\frac{1}{j\lambda })e^{\frac{j2\pi r}{\lambda }}\\ r=\sqrt{{\left(x-x_{i}\right)}^{2}+{\left(y-y_{i}\right)}^{2}+{\left(z-z_{i}\right)}^{2}} \end{array}\right.$$

where *l* indicates the *l*-th layer of the diffractive neural network, *i* represents the *i*-th neuron of the *l*-th layer, *r* denotes the Euclidean distance between the *i*-th neuron of the l-th layer and the (*l* + 1)-th layer, *j* is the imaginary unit $\sqrt{-1}$, λ represents the wavelength of the incoming wave, and $w_{i}^{l}(x,y,z)$ is the complex-valued field generated at point (x,y,z) of an neuron in layer (*l* + 1)-th by the *i*-th neuron located at point $\left(x_{i},y_{i},z_{i}\right)$ in layer *l*-th. The input plane is designated as 0-th layer. As a secondary wave source, the amplitude and relative phase of the secondary waves generated by the neuron are determined by the incident wave and the transmission function of the neuron. The output light fields of the *i*-th neurons in the *l*-th layer can be expressed as follows:

(2) $$Y_{i}^{l}(x,y,z)=w_{i}^{l}(x,y,z)\cdot t_{i}^{l}(x_{i},y_{i},z_{i})\cdot \sum_{k}Y_{k}^{l-1}(x_{i},y_{i},z_{i})=w_{i}^{l}(x,y,z)\cdot \left|A\right|\cdot e^{j△\theta }$$

where $t_{i}^{l}$ is the transmission function of the neuron, and the input at the *i*-th neuron of the *l*-th layer is the superposition of all neurons’ outputs from the previous (*l*-1)-th layer, which is denoted as $\sum_{k}Y_{k}^{l-1}(x_{i},y_{i},z_{i})$. $\left|A\right|$ represents the output amplitude of the *i*-th neuron, and Δ*θ* denotes the phase delay produced by the input light wave at the *i*-th neuron. The transmission coefficient of light can be expressed as follows:

(3) $$t_{i}^{l}(x_{i},y_{i},z_{i})=a_{i}^{l}(x_{i},y_{i},z_{i})e^{j\phi_{i}^{l}(x_{i},y_{i},z_{i})}$$

where $a_{i}^{l}(x_{i},y_{i},z_{i})$ indicates the neuron’s amplitude, and $\phi_{i}^{l}(x_{i},y_{i},z_{i})$ represents the neuron’s phase. In practical fabrication, the chosen substrate is often optically transparent with constant transmittance. The neurons processed on such a substrate have a constant amplitude, and this type of D^2^NN is referred to as phase-only D^2^NN.

However, training with the aforementioned integral formula consumes significant computational time. By introducing plane wave angular spectrum [47] and fast Fourier transform algorithms [48] during the training process, the computation in the spatial domain can be converted to the frequency domain, significantly reducing the computational load and thereby decreasing training time. In this paper, D^2^NN architecture is modeled using Python (v3.8.18) and the Pytorch (v1.8.1) framework. It runs on a server equipped with an NVIDIA A40 GPU, an AMD EPYC 7513 @2.6 GHz CPU, and 1024 GB RAM, operating on Windows 10 (Microsoft, Redmond, WA, USA). In our numerical analyses, firstly, it is necessary to assign an initial phase distribution to each diffraction layer. We employed the Xavier initialization method [49], which is a prevalent technique for neural network weight initialization. This method effectively prevents issues such as vanishing and exploding gradients by ensuring that the outputs at each layer maintain an appropriate variance, thereby enhancing the convergence speed during training and the generalization capability of the network. Next, we use the mean squared error function as the loss function, which is commonly utilized in D^2^NN algorithm. The training batch size is set to 16, with a learning rate of 0.001. Each layer comprises 32 × 32 neurons, and the phase parameters of each neuron are iteratively updated using the stochastic gradient descent algorithm to minimize the loss function. It should be noted that the nonlinear function sigmoid $2\pi /(1+e^{-\phi })$ is used to keep the phase ϕ of each update between 0 and 2π during the training.

**Figure 2:**
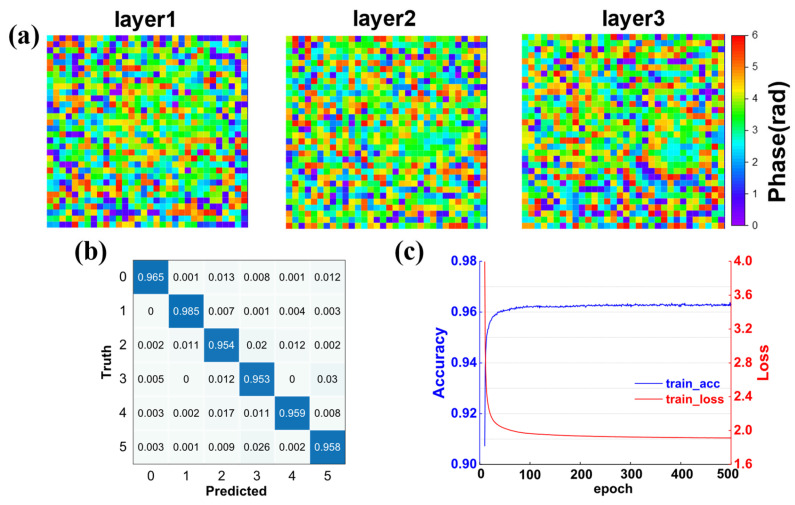
Numerical simulation results of D^2^NN. (**a**) Phase distribution of the hidden layers obtained by the training results of D^2^NN model. (**b**) Confusion matrix of the classification task on D^2^NN model in the final numerical simulation, achieving an overall accuracy of approximately 96.2% for six-class digit image classification. (**c**) The evolution of accuracy and loss value during the classification process with increasing the numbers of iteration.

The D^2^NN is trained using a dataset of 36,000 samples, with an additional 6030 samples reserved for testing. After that, we conduct the training of 500 epochs for the entirely phase-only three-layer D^2^NN to ensure sufficient learning and convergence of the network. Figure 2a illustrates the learned phase profile of three diffraction layers, while Figure 2b demonstrates the confusion matrix for the D^2^NN model, achieving an overall accuracy rate of approximately 96.2% in classifying six types of digit patterns. Figure 2c depicts the changes in loss value and accuracy during the training process; it is shown that the convergence speed of the model is faster in the training process, which indicates that the model training has been effectively optimized and adjusted.

### 2.2. All-Dielectric Metasurface Design

**Figure 3:**
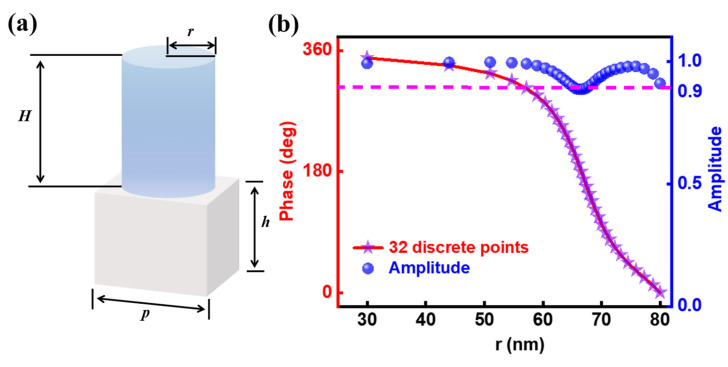
The geometry structure of unit cell of all-dielectric phased transmitarray. (**a**) Schematic diagram of a tunable transmissive meta-atom in an all-dielectric Huygens phase. Here, *H* = 180 nm, *h* = 350 nm, *p* = 360 nm. (**b**) A complete phase coverage from 0 to 2π for the cylinder with radius varies from 30 to 80 nm.

To perform a proof-of-concept demonstration of our diffractive image classifier framework, we designed a three-layer phased transmitarray to meet the requirement learned by D^2^NN algorithm. The meta-atom used to simulate neurons in the deep learning algorithm is shown in Figure 3a. It is composed of a silicon dioxide substrate with a periodicity of *p* = 360 nm and height *H* = 180 nm, and a cylindrical silicon nanodisk of height *h* = 350 nm is placed on top of it. Each metasurface consists of 1024 unit cells, each of which has a size of 360 nm × 360 nm × 530 nm. Figure 3b illustrates a complete phase coverage from 0 to 2π for a cylinder with the radius varying from 30 to 80 nm at a wavelength of 600 nm. For simplification in subsequent simulations, the continuous phase distribution from 0 to 2π was discretized into 32 points, each with a fixed phase difference of 11.25° from the neighboring locations [50]. The 32 discrete points are marked as star shapes on the graph, while the spherical markers indicate the amplitude of the unit cell corresponding to these 32 different radii, all of which are nearly greater than 0.9. Within the parameter range of this structure, the size of the silicon cylinder is smaller than the wavelength of the incident light, so the scattering of light is mainly dominated by the properties of a single scatterer and is not significantly affected by coupling between scatterers. At the same time, the design employs gradually varying scatterer sizes to ensure that the size changes between the adjacent scatterers are smooth, thereby avoiding abrupt changes and reducing scattering and diffraction losses of light. This local scattering effect and size design approach make light transmission and phase modulation more efficient, supporting the achievement of high transmittance [41,51].

## 3. Results

### 3.1. Digital Images Classification

**Figure 4:**
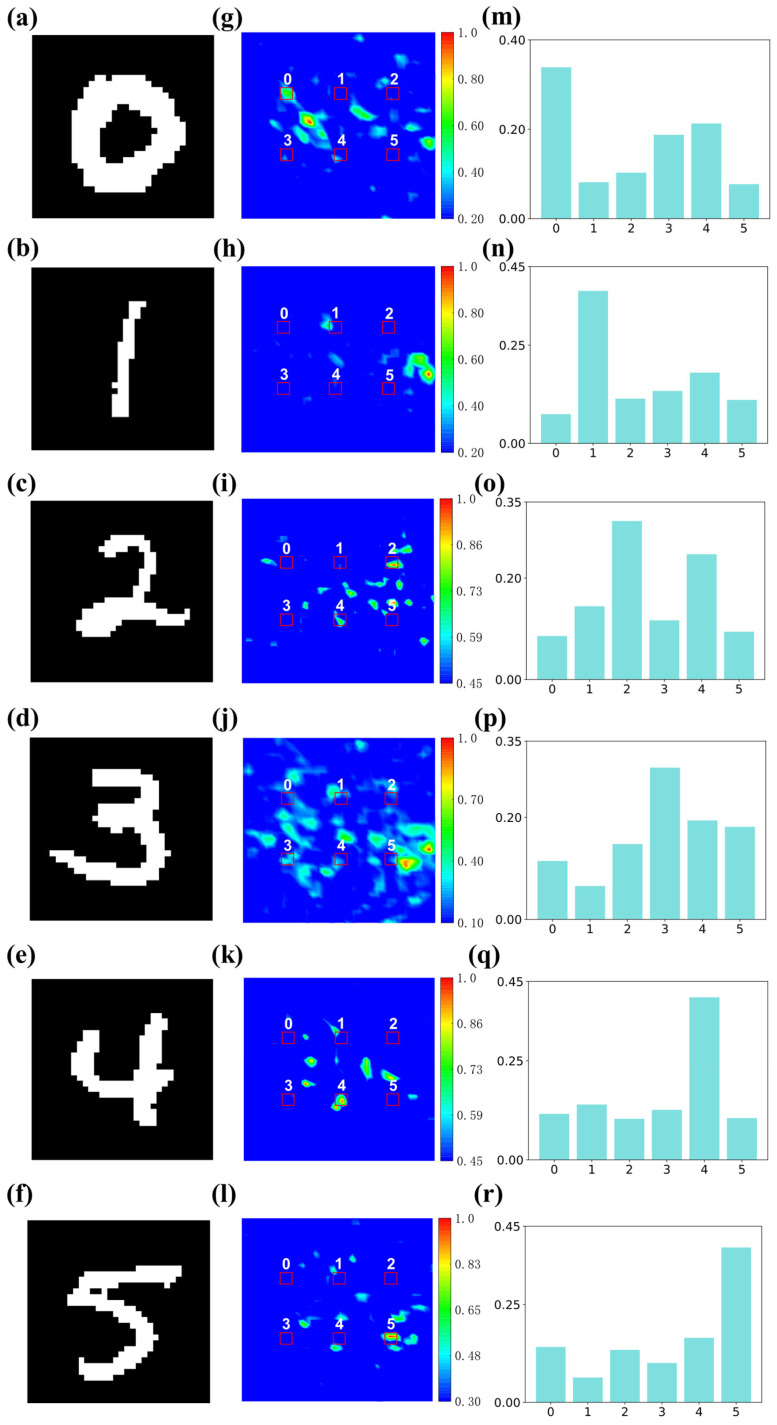
Schematic diagram of electromagnetic simulation samples and results. (**a**–**f**) Selected simulation test sample images. (**g**–**l**) Light field distribution images of simulation output planes. (**m**–**r**) Energy distribution graphs within each numerical label area.

To verify the role of the metasurface composed of the unit cell as a diffraction layer in the D^2^NN, the physical model was built in CST Studio Suite based on the conversion of the phase parameters of the training model into the structural parameters of the nanodisk. It was also necessary to properly set parameters such as frequency, plane wave, and boundary conditions. It took about 12 h to obtain the simulation results for the distribution of the output plane field for each sample. Figure 4a–f are the digital samples used for simulation, while Figure 4g–l depict the light intensity distribution on the detector plane for these test samples after electromagnetic simulation. On the detection plane, we arranged six 2 × 2 pixels as the target regions (the red rectangles in figures) corresponding to the six digits [52]. Figure 4m–r show the energy proportion within these six red rectangles. For every test sample, after passing through the three-layer D^2^NN, there was a target area within these six red rectangles that had the highest energy relative to the others, and the number represented by this area matched the corresponding label category in the algorithm. This demonstrates that the D^2^NN structure can accurately classify different handwritten digits with a processing speed close to the speed of light.

From the EM simulation results, it is shown that apart from the digit ‘3’, other digits had noticeable focal points in the target areas. It may have arisen from the relatively limited number of meta-atoms we used in the model since the total number of grids will increase exponentially with the increase in neurons, leading to a decrease in the overall network connectivity. This restriction reduces the network’s capacity to extract and convey image features precisely. By increasing the number of neurons in each layer and the number of diffraction layers, nonlinear functions can be more suitable for diffractive neural networks, thereby improving the recognition accuracy and recognizing more complex images. Alternatively, achieving fully connected topologies between the layers by optimizing the spacing of diffraction layers can ensure comprehensive neuronal diffraction interconnectivity thus guaranteeing effective information transmission between layers. From the perspective of a metasurface, one can choose a meta-atom capable of modulating both phase and amplitude simultaneously to achieve a more precise classification.

### 3.2. Animal Images Classification

**Figure 5:**
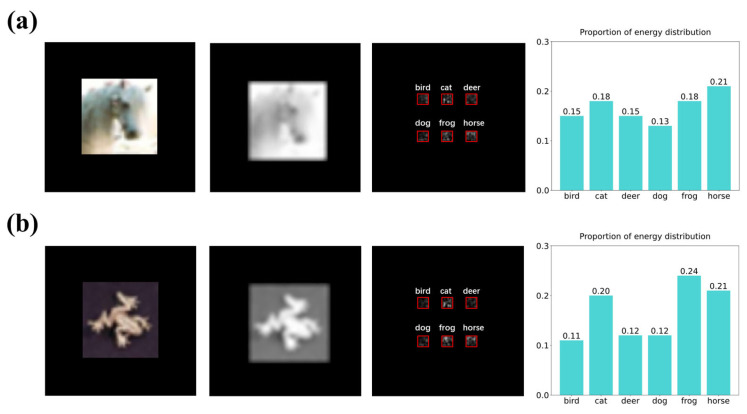
Illustrative diagrams of numerical simulation results for animal image samples. (**a**) The original image of samples marked as “horse”, the converted grayscale image, the light field distribution on the detector plane through five diffraction layers, and the energy proportion within each marked area on the detector plane. (**b**) The original image of samples marked as “frog”, the converted grayscale image, and the light field distribution on the detector plane through five diffraction layers, and the energy proportion within each marked area on the detector plane.

To study the applications on complex graphs, we also attempted to use the D^2^NN to identify and classify animal images. The samples came from the CIFAR-10 dataset; we selected six types of animals (including birds, cats, deer, dogs, frogs, and horses) to train the D^2^NN model. The dataset comprised three-channel RGB images, since D^2^NN is designed to handle monochromatic light, and we converted these images to grayscale during the preprocessing stage thus transforming them into one-channel images. Figure 5a,b show the original images and grayscale images of a horse and a frog, as well as the energy distribution map of the detector plane after D^2^NN processing. In order to classify those complex images, we expanded the size and increased the number of diffraction layers to 128 × 128 pixels and five layers, respectively, to enhance the computational capability of the D^2^NN. The numerical results show that more complex animal image classification can be successfully achieved. Due to the considerable noise and complexity of features in the images, the classification accuracy achieved by the model was relatively low for numerical images at only 55.1%. Next, we plan to integrate the optical 4*f* system and make improvements to the image preprocessing steps. Our aim is to better extract the image features fed into the D^2^NN and minimize noise interference thereby enhancing overall performance.

## 4. Conclusions

This research mainly demonstrates the design, numerical simulation, and full-wave electromagnetic validation of a phase-only all-optical D^2^NN based on an all-dielectric phased transmitarray, specifically in the performance of handwritten digit classification tasks. The detailed design process for the metasurface, along with the related numerical models and algorithms, is provided. The classification accuracy in numerical simulations reached about 90%. Selecting some digital samples from the dataset for full-wave electromagnetic simulations, which are consistent with the numerical simulation results, demonstrated the feasibility of D^2^NN in applications at the frequency of 600 nm.

## Figures and Tables

**Table 1:** Computational performance of different D^2^NN models on MNIST dataset.

Model	Network Size	Pixel Size	Incident Wavelength	Accuracy (%)
DONNs [36]	100 × 100 × 5	10 μm × 10 μm	700 nm	97.54
Multi-wavelength D^2^NNs [37]	200 × 200 × 5	4 μm × 4 μm	400 nm, 500 nm and 700 nm	95.6
SL-DNN [38]	200 × 200 × 1	8 μm × 8 μm	515 nm	97.08
Visible Light D^2^NN [39]	1000 × 1000 × 5	4 μm × 4 μm	632 nm	91.57
R-ODNN [40]	120 × 120 × 3	1 μm × 1 μm	1550 nm	94.46

## Data Availability

The original contributions presented in the study are included in the article, further inquiries can be directed to the corresponding authors.
